# Evaluating the Efficacy of a Guided and Unguided Internet-Based Self-help Intervention for Chronic Loneliness: Protocol for a 3-Arm Randomized Controlled Trial

**DOI:** 10.2196/36358

**Published:** 2022-07-22

**Authors:** Noëmi Seewer, Andrej Skoko, Anton Käll, Gerhard Andersson, Maike Luhmann, Thomas Berger, Tobias Krieger

**Affiliations:** 1 Department of Clinical Psychology and Psychotherapy University of Bern Bern Switzerland; 2 Department of Behavioural Sciences and Learning Linköping University Linköping Sweden; 3 Center for Social and Affective Neuroscience Linköping University Linköping Sweden; 4 Department of Clinical Neuroscience Karolinska Institute Stockholm Sweden; 5 Faculty of Psychology Ruhr University Bochum Bochum Germany

**Keywords:** loneliness, subjective social isolation, internet-based intervention, self-help, guidance, online, mobile phone

## Abstract

**Background:**

Loneliness, or perceived social isolation, is prevalent in both the general population and clinical practice. Although loneliness has repeatedly been associated with mental and physical health, research on interventions that reduce loneliness effectively is still rather scarce.

**Objective:**

This study aims to evaluate the efficacy of a guided and an unguided version of the same internet-based cognitive behavioral self-help program for loneliness (SOLUS-D) for adults.

**Methods:**

A total of 250 participants will be randomly assigned to 1 of 2 intervention groups (SOLUS-D with guidance or SOLUS-D without guidance) or a wait-list control group (2:2:1 allocation ratio). Adult participants experiencing high levels of loneliness will be recruited from the general population. Individuals currently experiencing at least moderately severe depressive symptoms, an ongoing severe substance use disorder, previous or current bipolar or psychotic disorder, or acute suicidality will be excluded from the trial. Assessments will take place at baseline, 5 weeks (midassessment), and 10 weeks (postassessment). The primary outcome is loneliness assessed using the 9-item University of California, Los Angeles Loneliness Scale at the posttreatment time point. Secondary outcomes include depressive symptoms, symptoms of social anxiety, satisfaction with life, social network size, and variables assessing cognitive bias and social behavior. The maintenance of potentially achieved gains will be assessed and compared at 6 and 12 months after randomization in the 2 active conditions. Potential moderators and mediators will be tested exploratorily. Data will be analyzed on an intention-to-treat basis.

**Results:**

Recruitment and data collection started in May 2021 and are expected to be completed by 2022, with the 12-month follow-up to be completed by 2023. As of the time of submission of the manuscript, 134 participants were randomized.

**Conclusions:**

This 3-arm randomized controlled trial will add to the existing research on the efficacy of loneliness interventions. Furthermore, it will shed light on the role of human guidance in internet-based treatments for individuals with increased levels of loneliness and the possible mechanisms of change. If SOLUS-D proves effective, it could provide a low-threshold, cost-efficient method of helping and supporting individuals with increased levels of loneliness.

**Trial Registration:**

ClinicalTrials.gov NCT04655196; https://clinicaltrials.gov/ct2/show/NCT04655196

**International Registered Report Identifier (IRRID):**

DERR1-10.2196/36358

## Introduction

### Background

Loneliness, or perceived social isolation, is a common phenomenon observed in clinical practice and in the general population. Some authors have even considered it an epidemic phenomenon [1]. Although an increase in the prevalence of loneliness has been observed after the onset of the COVID-19 pandemic and the accompanying social distancing measures [2,3], loneliness was a prevalent phenomenon even before. The prevalence of loneliness varies substantially among European countries [4]. In the general German population, 5% to 10% of individuals frequently experience feelings of loneliness [2,5], and a recent cross-temporal meta-analysis revealed an increase in loneliness in emerging adults over the past 40 years [6]. Within a representative German adult sample, loneliness was prevalent in all age groups, with peaks in young adults (aged approximately 30 years), in adults aged approximately 60 years, and in the oldest adults (≥80 years) [7]. Numerous studies have shown negative physical and mental health consequences associated with loneliness [8], and it is associated with all-cause mortality [9,10]. Consequently, loneliness has recently been increasingly recognized as a public health concern that needs to be addressed, leading to several national initiatives (eg, in the United Kingdom and Japan) to tackle loneliness and objective social isolation on a societal level. Furthermore, attempts have been made to develop and evaluate interventions to reduce loneliness at the individual level. However, evidence on what interventions work and for whom is still limited [11].

### Conceptualizing Loneliness

Human connection is regarded as a basic psychological need [12]. Consequently, when this need cannot be met, feelings of loneliness may arise. Peplau and Perlman [13] defined loneliness as an aversive subjective experience resulting from a discrepancy between actual and desired social relationships. The quantity and quality of social contact seem to be relevant in this regard [14]. This means that a person can feel lonely despite having many social relationships, whereas another person with only a few meaningful connections may not feel lonely. Therefore, it is essential to distinguish between loneliness (ie, subjective social isolation) and objective social isolation. Although loneliness is often experienced as stressful, perceived control over the frequency with which individuals socialize, and the amount of time spent alone play an important role in whether social isolation, living alone, or being alone is perceived as distressing [7]. Researchers have argued that it is necessary to distinguish between different forms of loneliness in terms of the duration of the experience. For example, Young [15] suggested differentiating among transient, situational, and chronic loneliness. Transient feelings of loneliness are adaptive in motivating individuals to reconnect with others [16] and should not be considered pathological [17], whereas chronic loneliness is a more stable condition expressed as difficulties in engaging in satisfying social relationships. This experience is intrinsically aversive and is associated with a variety of severe consequences for physical and psychological health [18].

### Maintaining Factors of Chronic Loneliness

According to the evolutionary theory of loneliness [16], transient feelings of loneliness are adaptive by functioning as a signal comparable with hunger or thirst, motivating individuals to reconnect with others to increase the likelihood of survival. However, some people get stuck in a vicious circle that maintains feelings of loneliness, leading to chronic loneliness. Cacioppo et al [16,19] outlined a cognitive model of loneliness. The model indicates that perceived social isolation causes initial social withdrawal, allowing one to observe and evaluate immediate social situations through physical distance [20]. When perceiving oneself as socially isolated, the motive for self-preservation may increase, leading to heightened hypervigilance for potential social threats and attacks [21,22]. Consequently, individuals perceive social stimuli as more threatening, especially when a situation is neutral or ambiguous. In line with this, individuals with increased levels of loneliness have shown a negative bias in several phases of social information processing (see the study by Spithoven et al [23] for an overview). According to Cacioppo and Hawkley [19], counterproductive social behavior, such as social avoidance or preventive rejection of others, is reinforced by biased information processing. Such behavior may help prevent further rejection or attacks but may also hinder behavior that could promote close satisfying social connections. In addition, the results of several studies show that individuals with chronic loneliness may lack social skills, such as authenticity [24] or self-disclosure [25,26], expressing emotions [27], or being compassionate toward others and the self [28], which are important in building close relationships [29-34]. Heightened rejection sensitivity [22] and negative evaluations of others can further foster negative experiences and expectations of social interactions [35]. Hence, a lack of perceived social efficacy, (ie, confidence in the ability to engage in social interactions or initiate and maintain interpersonal relationships) can be reduced in individuals with increased levels of loneliness [22,36,37]. The resulting social behavior hampers the forming of new relationships and the deepening of existing relationships, leading to more negativity and stronger feelings of loneliness. Consequently, negative biases in social situations are further increased, leading to a vicious circle and, therefore, to chronic loneliness.

### Loneliness and Mental Disorders

Loneliness can be viewed as a transdiagnostic phenomenon [38], which is frequent in various psychological disorders and psychopathological symptoms. For example, it has been found to be associated with depression [39], psychosis [40], suicidal ideation [41,42], and generalized anxiety [5]. Impaired sleep quality and insomnia symptoms have also been repeatedly reported in individuals with increased levels of loneliness [43]. In line with these findings, loneliness is associated with reduced well-being [44]. Owing to the cross-sectional nature of most studies, the causality for the associations between loneliness and mental health problems is often unclear. However, reciprocal relationships have been found for a variety of psychopathological symptoms, such as depressive symptoms and symptoms of social anxiety [45-47]. Therefore, loneliness can be considered a risk and maintaining factor of mental disorders.

### Interventions Against Loneliness

Loneliness, with its many detrimental effects on health, has led researchers to develop various interventions aiming to alleviate the condition. In general, psychological interventions have been found to reduce loneliness [48]. In an attempt to categorize different approaches, Masi et al [49] identified four groups of interventions that have been studied for loneliness: (1) developing social skills, (2) increasing social support, (3) augmenting opportunities for social interaction, and (4) changing maladaptive social cognitions. Interventions focusing on and changing social cognition were shown to be the most promising approaches compared with other intervention types [49]. A more recent systematic review [11] corroborated these findings by concluding that the most promising individual interventions had focused on cognitive interventions. These results are in line with the abovementioned cognitive model of loneliness, according to which interventions need to address hypervigilance to social threats and related cognitive biases that characterize individuals with increased levels of loneliness [16,19]. Recently, there have been several systematic review updates on interventions against loneliness in older adults in general [50], in individuals with mental health problems [51], and in randomized controlled trials (RCTs) [38], with all 3 reviews highlighting the largely unmet need for high-quality research on psychological interventions for loneliness.

### Internet-Based Interventions for Loneliness

In recent years, cognitive behavioral therapy (CBT) has been applied as internet-delivered CBT (ICBT). Location- and time-independent use, high degree of anonymity and privacy, and low costs because of easy scalability are some of the advantages of ICBT [52]. Although most people are familiar with feelings of loneliness, it often has a negative connotation and is stigmatized [53]. Thus, low-threshold access to interventions may be especially helpful for individuals with increased levels of loneliness in need. In addition, it has been argued that ICBT might especially be accepted by individuals with increased levels of loneliness showing avoidant and withdrawing social behavior [54], as internet-based interventions are associated with diminished anxiety about social interactions with therapists [55].

Recently, there have been some promising studies on low-threshold internet-based self-help interventions against loneliness. In a pilot RCT, Käll et al [54] compared a guided ICBT with a wait-list control group. Significant reductions in loneliness were found with a between-group effect size (Cohen *d*) of 0.77 at the postintervention time point [54]. A further decrease in loneliness was observed at the 2-year follow-up [56].

More evidence for effectively alleviating loneliness by means of ICBT stems from a 3-armed trial comparing 2 active intervention groups (ICBT vs internet-based interpersonal therapy [IIPT]) with a wait-list control group [57]. Loneliness in individuals in the ICBT condition was significantly reduced after the intervention phase, with a moderate to large effect size (Cohen *d*=0.71) compared with the wait-list and a moderate effect size (Cohen *d*=0.53) compared with the IIPT group. No significant differences regarding loneliness were observed between the IIPT and wait-list groups [57].

An unguided, web-based, friendship enrichment program, including 3 coping strategies to tackle loneliness (ie, network development, adapting personal standards, and reducing the importance of the discrepancy between actual and desired relationships), was tested in 239 participants aged 50 to 86 years [58]. This study drew on the definition of loneliness from Peplau and Perlman [13] and suggested that not only increasing opportunities for social contact but also focusing on the discrepancy between actual and desired relationships and expectations for relationships are important in alleviating loneliness. On average, loneliness declined significantly over the course of the study [58]. However, the authors reported high dropout rates, with only 36% of the participants completing all modules of the program [59].

Although the first studies on ICBT for loneliness have shown promising results, more studies are needed for several reasons. First, the study designs applied in previous trials on ICBT for loneliness [54,57] do not allow controlling for nonspecific effects such as human contact on the outcome. However, guidance (ie, weekly human contact by email) might affect the decrease in loneliness other than through CBT by addressing principles relevant to building meaningful relationships (eg, validation) [54]. Moreover, guidance also yields larger effects when comparing guided and unguided interventions in other application fields of ICBT, such as depression [60]. Thus, it is important to investigate the effect of guidance on outcomes in internet interventions for loneliness. Second, there is a gap in the literature on who can profit from ICBT for loneliness and, for example, whether some people profit more from a guided than from an unguided version. Third, little is known about how interventions for loneliness work, and research is needed to identify mechanisms of change in these interventions and, thereby, improve the understanding of chronic loneliness.

### Objectives

Given the need for more high-quality RCTs of interventions for alleviating loneliness, this study will be conducted to investigate the following objectives. First, we will test the efficacy of 2 web-based interventions (ICBT with or without guidance) compared with a wait-list control group regarding loneliness (primary outcome) and a range of secondary outcomes (eg, depression, anxiety, satisfaction with life, and factors of the cognitive model of loneliness). We expect individuals in both intervention groups to show a greater reduction in loneliness and more pronounced effects in the secondary outcomes than in the wait-list control group.

Second, we will compare the efficacy and long-term effects of the 2 active interventions regarding primary and secondary outcomes to specify the effects of added human contact through guidance. We expect greater changes in the primary and secondary outcomes in the intervention with guidance than in the intervention without guidance.

Third, we will test potential baseline predictors for the efficacy of the interventions and explore moderators between the 2 active conditions to better understand which intervention (with or without guidance) works best for whom. Considering the exploratory nature of this objective, we have no specified hypothesis for this objective.

Finally, we will explore potential mediators (mechanisms of change) of the interventions. We expect changes in cognitive bias and counterproductive social behavior, as described in the cognitive model of chronic loneliness [19], to explain reductions in loneliness.

## Methods

### Study Design

**Figure 1:**
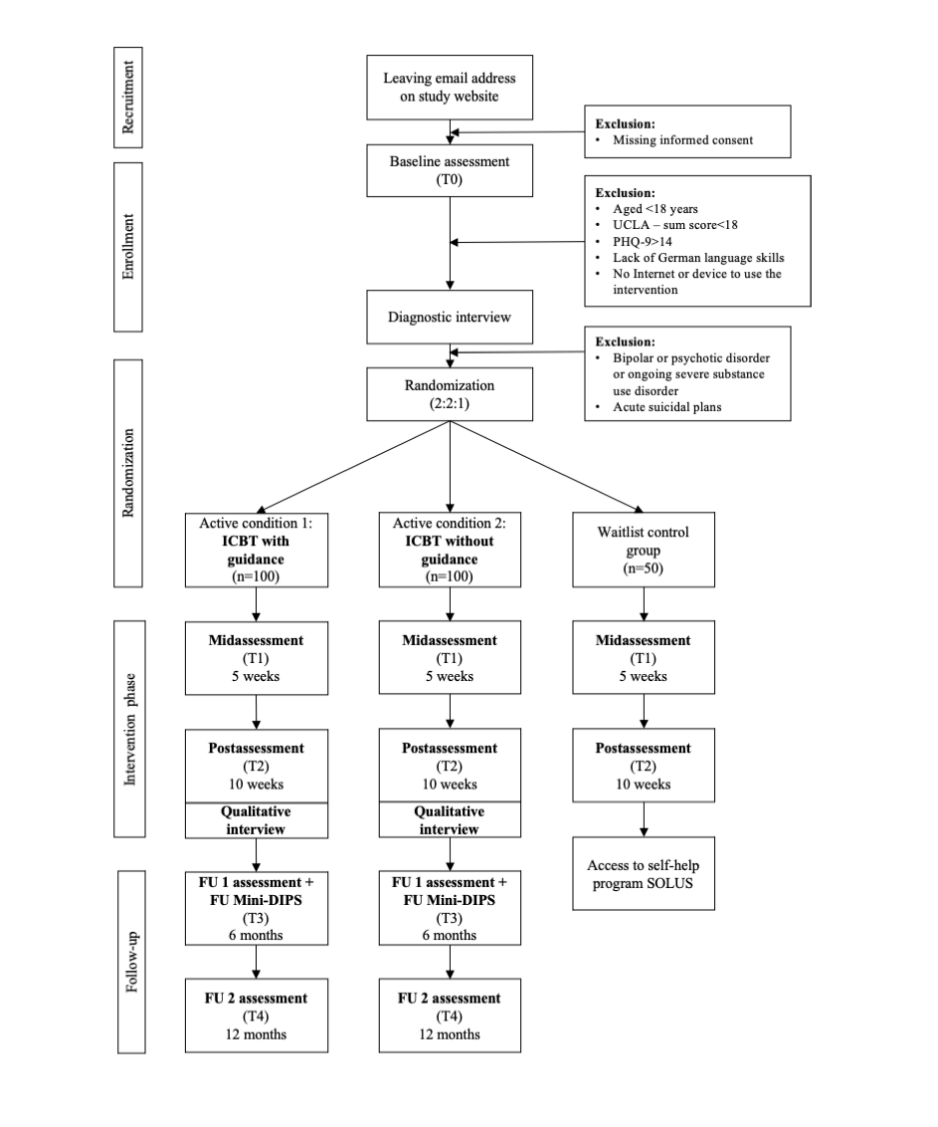
Participant flow. FU: follow-up; ICBT: internet-based cognitive behavioral therapy; Mini-DIPS: Mini Diagnostic Interview for Mental Disorders; PHQ-9: Patient Health Questionnaire-9; T0: time point 0; T1: time point 1; T2: time point 2; T3: time point 3; T4: time point 4; UCLA: University of California, Los Angeles.

This study uses a 3-arm RCT design to compare 2 intervention groups (SOLUS-D with individualized guidance by a coach and SOLUS-D without guidance), with a wait-list control group. Participants in the 2 active conditions will have access to the 10-week internet-based self-help intervention immediately after randomization. Participants in the wait-list control group will receive access to the program after the intervention phase of 10 weeks and the completion of the postassessment measure. Assessments for all participants will be administered at baseline (time point 0), 5 weeks (time point 1, midtreatment), and 10 weeks (time point 2, after treatment) after randomization. In addition, participants in the 2 intervention groups will be followed up at 6 (time point 3) and 12 months (time point 4) after randomization. As participants in the control group will have access to the program after 10 weeks for ethical considerations, they will not be included in the follow-up measurements. The study design is illustrated in Figure 1.

### Ethics Approval

This study is being conducted in compliance with the Declaration of Helsinki and has been approved by the Cantonal Ethics Committee, Bern (ID:202-01298). All participants will receive written information about the aim of the study, benefits, risks of participation, and procedure of the study. Participants will be informed that they can withdraw from the study at any time without disclosing their reasons. Written informed consent will be obtained from all participants before the baseline assessment. The trial was preregistered with ClinicalTrials.gov (NCT04655196).

### Inclusion and Exclusion Criteria

The inclusion criteria for this study are as follows: (1) age of at least 18 years; (2) a score of at least 18 points on the University of California, Los Angeles (UCLA) Loneliness Scale-9; (3) sufficient German language skills; (4) access to an internet connection and a device to use the intervention; and (5) signed informed consent and the provision of a contact in case of emergency. The cutoff score of the UCLA Loneliness Scale-9 was derived from the cutoff score used in the aforementioned study by Käll et al [54] by transforming it to a mean score and adapting it to the short version used in this study. Individuals will be excluded from the study if they (1) currently have at least moderately severe depressive symptoms (as indicated by a Patient Health Questionnaire-9 [PHQ-9] score >14), (2) have a lifetime diagnosis of schizophrenia or bipolar disorder, (3) fulfill the criteria for a severe substance use disorder, or (4) have acute suicidal plans. The exclusion criterion for schizophrenia will be assessed by means of screening questions regarding a former formal diagnosis or by the diagnostic interview Mini Diagnostic Interview for Mental Disorders (Mini-DIPS). The exclusion criteria for substance use disorder and acute suicidal plans will be assessed during the diagnostic interview (Mini-DIPS). In this study, there will be no restrictions regarding the use of other treatments during the study.

### Participants, Recruitment, and Randomization

Participants will be recruited in German-speaking countries through reports in newspapers and radio interviews, internet forums, social media, our study website [61], and the website for ongoing studies from our research hub [62]. After checking the inclusion and exclusion criteria by means of the baseline assessment and a diagnostic interview, eligible participants will be automatically randomly allocated in a blockwise manner (blocks of 10 participants) on the web-based platform Qualtrics (Qualtrics XM) to either of the 2 active conditions or the wait-list control group (randomization ratio 2:2:1).

### Procedure

Interested individuals will leave their email addresses on the study website and receive the study information and consent form by email. Participants will be invited to ask questions about the study by phone. Once the signed consent form is returned, a link to the baseline assessment will be sent via email. After the baseline assessment is completed, the diagnostic interview will be conducted over the telephone by trained master’s students and members of the study team. Subsequently, eligible participants will be randomly allocated to 1 of the 3 study groups. After randomization, individuals randomized to the 2 active conditions will receive immediate access to the internet-based self-help program SOLUS-D. Individuals in the wait-list control group will receive access to the program after a waiting period of 10 weeks after randomization. Participants in all 3 conditions will be asked to complete additional questionnaires 5 and 10 weeks after randomization. Furthermore, individuals in the 2 active conditions will be followed up for 6 and 12 months after randomization. Participants will receive a reminder via email each week for up to 3 weeks if they do not complete the questionnaires. After completing the postassessment measure, a random subsample of individuals in the active condition will be asked to participate in a qualitative interview. The aim is to gain more profound insight into their experience with the program and possible adverse effects during the intervention phase. At 6 months after randomization, participants in the 2 active conditions will be contacted once more by phone to conduct a second diagnostic interview. After the intervention phase, all participants will continue to have access to the self-help intervention in an unguided format. The participants will not be compensated for partaking in the trial.

### Intervention: Internet-Based Self-help Program—SOLUS-D

Participants in both active conditions will have access to SOLUS-D, an internet-based self-help program. This intervention is a German adaptation and extension of the ICBT program developed by Käll et al [54]. The program is an internet-based and interactive self-help guide with text, audio, and video files and a diary function (Table 1). The program comprises 9 modules based on CBT principles. Expanding on the original program of Käll et al [54], the program used in this study has been enriched and extended with elements of mindfulness, self-compassion, acceptance and commitment, and social skills by our team. We will recommend completing 1 module per week and that participants work through the program sequentially. Each module builds on the previous module and takes approximately 50 minutes to complete. Theoretically, all modules can be completed at once; thus, they are not gradually made available weekly. Participants can navigate the content of the completed modules as they want and repeat the exercises and modules. Apart from working on the modules, the participants will be asked to complete exercises and web-based diaries as often as possible. The program is accessible on any computer, tablet, or smartphone. Secure Sockets Layer encryption will be used to secure internet-based communication with the program and guides, and participants will be identified with anonymous log-in names and passwords. The platform on which the program runs has been successfully used for several research projects in our research unit [63,64].

**Table 1:** SOLUS-D content.

Module number	Module	Content	Exercises
1	Loneliness and personal values	Information about the program use, structure of the intervention, and psychoeducation on loneliness and personal values are provided in this module.	Vicious circle of loneliness Values in different life areas Introduction to mood diary (continuous exercise)
2	Goal setting and mindfulness	Personal goals are set and a theoretical and practical introduction to mindfulness is provided.	Setting goals Mindful breathing Body scan 3-minute breathing space
3	Self-compassion	A theoretical and practical introduction to self-compassion is provided.	Kindness meditation: self-compassion LKM^a^ Introduction (self-) compassion diary (continuous exercise)
4	Acceptance of loneliness and solitude	The importance of emotions is highlighted and a strategy for accepting emotions is introduced. Furthermore, time spent alone is reflected.	Accepting emotions Exposition with time spent alone Reframing time spent alone
5	Identifying and changing thoughts	The impact of negative automatic thoughts and the relationship among thoughts, experiences, and behavior are introduced. Dysfunctional thoughts are identified and revised.	Identifying NAT^b^ Challenging NAT and formulating alternative thoughts
6	Rumination and behavioral experiments	Strategies for dealing with rumination and the idea of behavioral experiments are introduced.	Disrupting rumination Behavioral experiments
7	Social relationships and feeling connected	The current social relationship situation is evaluated more closely, values in social relationships are identified, and various social skills for relationship building are introduced.	Social convoy Values in close relationships Boundaries
8	Building social activities	The relationship between behavior and loneliness is further highlighted, avoidance and passivity are addressed, and value-based social activities are introduced.	Avoidance and passivity Value-based behavioral activation
9	More social activities and further goals	Obstacles with behavioral activation are addressed, and new activities can be planned. Finally, the content of all modules is reviewed, and future goals can be set.	Value-based behavioral activation Formulating further goals Strategy toolbox

^a^LKM: Loving Kindness Meditation.

^b^NAT: negative automatic thought.

### Conditions

#### ICBT With Guidance

Individuals in this condition will use the SOLUS-D program while being guided by trained and supervised coaches. The coaches will regularly monitor the use of the program, provide weekly written feedback via chat within the self-help platform on exercises, and motivate participants to work with the program continuously. Each message will comprise personalized feedback on the participants’ work during the previous week and answers to their questions. The content of these messages will be semistructured and manualized according to the theoretical model of Supportive Accountability [65]. This model argues that adherence increases with human support through accountability to a coach. The coaches will be 2 psychologists with a master’s degree in clinical psychology in their first year of a postgraduate CBT program and several master’s students in their last term of a graduate program in clinical psychology. All coaches will be trained in the specific approach and supervised by NS and TK.

#### ICBT Without Guidance

In this condition, participants will also have access to SOLUS-D. However, they will not be guided and will use it on their own; yet, the participants will receive weekly automated and fully standardized emails during the 10 weeks. The content of these emails aims to remind and motivate participants to continue engaging with the program. Within the information about group allocation, it is explicitly stated that the automated email is automatically sent from a computer and not by a human being. During the intervention period, questions concerning technical issues with the program will be answered upon request by the study team.

#### Wait-list Control Group

Participants in this condition will receive access to the unguided intervention 10 weeks after randomization (ie, after the postassessment time point).

### Measures

#### Overview

Demographic information such as gender, marital status, and education level will be self-reported by participants at baseline. In addition, we will assess participants’ medication status at baseline and their use of psychotherapy at every measurement time point. Table 2 summarizes the instruments and schedule of the assessments in this study. In case there was no German version of a scale available, the original scale was translated from English to German by our research group and back translated by a native English-speaking person. Differences between this back-translation and the original scale were discussed until a consensus was reached regarding the necessary changes in the German version.

**Table 2:** Assessment timeline.

Type of variable and variable		Measurement	Baseline (time point 0)	5 weeks (time point 1)	10 weeks (time point 2)	6 months^a^ (time point 3)	12 months^a^ (time point 4)
**Primary outcome**							
	Loneliness	UCLA^b^ Loneliness Scale 9-item version	✓	✓	✓	✓	✓
**Secondary outcomes**							
	Depression	Patient Health Questionnaire-9	✓	✓	✓	✓	✓
	Social anxiety	Social Interaction Anxiety Scale and Social Phobia Scale	✓	✓	✓	✓	✓
	Satisfaction with life	Satisfaction with Life Scale	✓	✓	✓	✓	✓
	Social isolation	Social Network Index	✓		✓	✓	✓
	Self-compassion	Sussex Oxford Compassion Scale for the Self	✓		✓		
	Maladaptive personality traits	PID-5-BF+^c^	✓		✓		
	Misantropy	Bern Embitterment Inventory^d^	✓		✓		
	Motivation for solitude	Motivation for Solitude Scale–Short Form^d^	✓		✓		
	Negative effects	Inventory for the Assessment of Negative Effects of Psychotherapy^a^			✓		
	Patient satisfaction	Client Satisfaction Questionnaire^a^			✓	✓	
	System usability	System Usability Scale^a^			✓		
	Mental disorders	Mini Diagnostic Interview for Mental Disorders	✓			✓^a^	
**Mechanisms of change**							
	Interpretation bias	Interpretation and Judgmental Bias Questionnaire^e^	✓	✓	✓		
	Rejection sensitivity	Adult Rejection Sensitivity Questionnaire	✓	✓	✓		
	Social avoidance	Cognitive-Behavioral Avoidance Scale^d^	✓	✓	✓		
	Self-Disclosure	Distress Disclosure Index	✓	✓	✓		
	Authenticity	Kernis and Goldman Authenticity Inventory–Short Form	✓	✓	✓		
	Self-esteem	Rosenberg Self-Esteem Scale	✓	✓	✓		
	Therapeutic alliance	Working Alliance Inventory for internet interventions^a,f^		✓			
**Moderators**							
	Mobility	Patient Questionnaire for Medical Rehabilitation^d^	✓				
	Attachment style	Adult Attachment Scale	✓				
	Childhood trauma	Childhood Trauma Questionnaire	✓				
	Demographic variables	N/A^g^	✓				

^a^Intervention groups only.

^b^UCLA: University of California, Los Angeles.

^c^PID-5-BF+: Personality Inventory for the Diagnostic and Statistical Manual of Mental Disorders-5 Brief Form Plus.

^d^Only subscales.

^e^Interpretation bias only.

^f^A total of 4 items will not be presented to participants in the SOLUS-D+automated feedback group as they are not plausible (eg, “The coach really cares about my well-being”).

^g^N/A: not applicable.

#### Primary Outcome Measure

Loneliness, assessed with the 9-item short version [66] of the Revised UCLA Loneliness Scale [67,68] at the postassessment time point, is the primary outcome. The original scale, comprising 20 items, assesses 3 different facets of loneliness: intimate loneliness, relational loneliness, and collective loneliness [69]. The short version comprises the 3 items with the highest loading on each factor [70]. The reliability and validity of this short version are comparable with those of the full 20-item version [70]. The response options are *never* (1), *rarely* (2), *sometimes* (3), and *always* (4). Ratings are summed, and scores range from 9 to 36, with higher scores indicating greater loneliness levels.

Previous studies revealed a discrepancy, for example, in the prevalence of loneliness, when assessing loneliness directly or indirectly [71]. Therefore, loneliness will be further assessed with a single direct question (“Do you feel lonely”; rated on a 4-point scale with the response options 0=*no, never;* 1=*yes, sometimes*; 2=*yes, quite often*; 3=*yes, very often*) and a 3-item very short version [72] of the UCLA Loneliness Scale for which German population norms exist [73].

#### Secondary Outcome Measures

*Depressive symptoms* will be assessed using the 9-item depression module of the PHQ-9 [74,75]. All 9 items correspond to the 9 Diagnostic and Statistical Manual of Mental Disorders (DSM)–IV criteria for depression. The items are rated on a 4-point scale from 0 (not at all) to 3 (nearly every day). Ratings are summed up, and scores range from 0 to 27. The PHQ-9 shows good validity [74] and sensitivity to change [75].

*Symptoms of social anxiety* will be assessed with the short forms of the Social Interaction Anxiety Scale and Social Phobia Scale [76]. These 2 scales complement each other and are mostly administered together. The 12 items are rated on a 5-point scale from 0 (not at all) to 4 (extremely). The Social Interaction Anxiety Scale and Social Phobia Scale showed good validity and sensitivity to change over time [76].

*Satisfaction with life* will be measured with the Satisfaction With Life Scale [77,78]. A total of 5 items, such as “In most ways my life is close to my ideal,” are rated on a 7-point scale from 1 (strongly disagree) to 7 (strongly agree). This scale shows good psychometric properties and norm values based on a large German sample [79].

*Self-esteem* will be assessed using the 10-item revised German version [80] of the Rosenberg Self-Esteem Scale [81]. This scale measures positive and negative aspects of self-esteem. Items are rated on a 4-point Likert scale ranging from 0 (strongly agree) to 3 (strongly disagree). The internal consistency of the 1 factorial solution is good (Cronbach α=.84) [80].

*Objective social isolation* will be measured using the Social Network Index [82]. This scale comprises 12 items assessing 12 different types of social relationships (eg, partner, parents, children, other close family members, close neighbors, friends, and fellow volunteers). Participants will be asked how many relationships of each type they have and how many of them they are in contact with at least once every 2 weeks. The network size, network diversity, and number of embedded subnetworks can be calculated using the Social Network Index [83].

*Maladaptive personality traits* will be assessed using the 34-item Personality Inventory for the DSM-5 Brief Form Plus [84], a 34-item short version of the Personality Inventory for DSM-5 [85]. The Personality Inventory for DSM-5 Brief Form Plus assesses 5 domains and 15 facets according to criterion B of the Alternative Model of Personality Disorders included in DSM-5, which are Negative Affectivity, Detachment, Antagonism, Disinhibition, and Psychoticism, plus the International Classification of Diseases (ICD)-11 domain Anankastia comprising 2 facets. Items are rated on a 4-point response scale ranging from 0 (very false or often false) to 3 (very true or often true). Scores can be calculated for the 17 facets and the 6 domains. Good internal consistency has been shown for the domain trait scores (McDonald ω=0.81) [84]. The average score of these 6 maladaptive trait domains can be used as an indicator of the severity of personality dysfunction according to the DSM-5 section III and the ICD-11 classification of Personality Disorders [86].

*The negative effects of the intervention* will be assessed with the Inventory for the Assessment of Negative Effects of Psychotherapy (INEP) [87]. The INEP assesses any adverse effects on social, intrapersonal, or work-related situations and whether they are attributed to the intervention. As in other studies on internet-based interventions, the INEP was slightly adapted for its use with internet-based interventions.

*Client satisfaction with the treatment* will be assessed with the Client Satisfaction Questionnaire-8 [88]. The 8 items are rated on a 4-point scale from 1 (low satisfaction) to 4 (high satisfaction). We adapted this measure to explore participants’ satisfaction with the internet intervention applied in this study. The Client Satisfaction Questionnaire-8 is a valid measure for assessing client satisfaction [88] and shows good internal consistency (Cronbach α=.90) [89].

*The usability* of the web-based program will be assessed using the System Usability Scale [90]. For this study, we adapted the measure to explore the experienced usability of the web-based program used in this trial. A total of 10 items are rated on a 5-point scale ranging from 0 (*strongly disagree*) to 4 (strongly agree). The usability score is obtained by multiplying the sum of all item scores by 2.5 and ranges from 0 to 100.

*Diagnoses of mental disorders* will be assessed using the short version of the Mini-DIPS–Open Access [91]. This structured interview is openly accessible and allows the reliable assessment of diagnoses according to the DSM-5 and ICD-10.

*Adherence* to the web-based program will be assessed using different indicators, such as (1) the number of modules completed and (2) time spent in the program. A module is completed when each page per module has been clicked at least once.

#### Assessment of Potential Mechanisms of Change

*Interpretation bias* will be assessed using the corresponding subscale of the Interpretation and Judgmental Bias Questionnaire [92,93]. The Interpretation and Judgmental Bias Questionnaire is a 24-item scale comprising brief scripts of 20 social events and 4 nonsocial control events. The social events can be divided into ambiguous, mildly negative, profoundly negative, and positive social events. For this study, we only assessed interpretation and not judgmental bias. For this purpose, each script will be followed with 4 alternative answers. The answers reflect positive, neutral, mildly negative, or profoundly negative interpretations of the event. The participants will be asked to rank the probability of these 4 interpretations. The score is the mean rank given to the profoundly negative interpretation of the scenarios and ranges between 1 and 4. A lower score indicates more negatively biased processing [94].

*Rejection sensitivity* will be assessed using the adapted adult version (Adult Rejection Sensitivity Questionnaire) [95] of the Rejection Sensitivity Questionnaire [96]. In the Adult Rejection Sensitivity Questionnaire, 9 hypothetical interpersonal situations are presented, and respondents indicate how they would feel or think in the stated situations. Respondents indicate on a 6-point scale how concerned they would be in that situation (very unconcerned to very concerned) and how likely they would expect to be accepted (very unlikely to very likely). One study shows high internal consistency (Cronbach α=.87) for the total score in a sample of adults with borderline personality disorder and acceptable consistency (Cronbach α=.75) for a healthy sample of adults [97].

*Social avoidance behavior* will be assessed with the subscale Behavior-social avoidance of the Cognitive-Behavioral Avoidance Scale [98,99]. Participants are asked to rate their social behavior on a 5-point Likert scale ranging from 1 (not at all true for me) to 5 (extremely true for me; eg, “I make excuses to get out of social activities.”). The subscale has shown good internal consistency (Cronbach α=.86) [99].

*Comfort with self-disclosure* will be assessed using the Distress Disclosure Index [100]. It is a 12-item scale designed to measure the degree to which a person is comfortable talking with others about personally distressing information (eg, “I am willing to tell others my distressing thoughts”). Items are rated on a 5-point Likert-type scale, with responses ranging from 1 (strongly disagree) to 5 (strongly agree). The Distress Disclosure Index has shown good psychometric properties [101].

*Authenticity* will be assessed using the 20-item short form (Kernis-Goldman Authenticity Inventory [KGAI]–Short Form [102]) of the KGAI version 3 [103]. It assesses 4 underlying dimensions of authenticity (awareness, unbiased processing, behavior, and relational orientation), and items are rated on a 5-point Likert scale ranging from 1 (*strongly disagree*) to 5 (*strongly agree*). The KGAI–Short Form shows good internal consistency (Cronbach α=.87) and good convergent and discriminant validity [102].

*Self-compassion* will be assessed using the Sussex Oxford Compassion Scale for the Self [104]. This scale measures five dimensions of compassion: (1) recognizing suffering, (2) understanding the universality of suffering, (3) feeling for the person in suffering, (4) tolerating uncomfortable feelings, and (5) motivation to act to alleviate the suffering. The 20 items will be rated on a 5-point scale ranging from 1 (not at all true) to 5 (always true). This scale shows adequate internal consistency and convergent and discriminant validity [104].

*Misanthropy* will be assessed using the respective subscale of the Bern Embitterment Inventory [105]. This subscale measures a general contempt of human beings (eg, “Sometimes I feel hatred towards mankind or a part of it”). The 4 items are rated on a scale from 0 (I do not agree) to 4 (I agree). The Cronbach α for this scale is .65 [105].

*Self-determined motivation for solitude* will be assessed with the corresponding 8-item subscale from the Motivation for Solitude Scale–Short Form [106]. This subscale measures the degree of the desire to spend time alone. Items are introduced with “When I spend time alone, I do so because...” and rated on a 4-point Likert scale from 1 (not at all important) to 4 (very important). The answers are summed, and higher scores indicate a higher self-determined motivation for solitude. The Motivation for Solitude Scale–Short Form is a reliable and valid measure of the motivation for solitude [106].

*Working alliance* will be assessed with the Working Alliance Inventory (Working Alliance Inventory-Short Revised) [107] adapted for guided internet interventions (WAI-I) [108]. The WAI-I comprises two subscales: (1) task and goal agreement dimension and (2) bond with the guide dimension (bond). The full scale comprises 12 items (eg, “With the online program, it has become clearer to me how I can change”) that are answered on a 5-point Likert scale ranging from 1 (never) to 5 (always). Participants in the ICBT condition without guidance will only respond to the 8 items of the task and goal dimension as the items of the bond dimension are not plausibly answerable. The WAI-I is a valid instrument for measuring working alliance in the context of guided interventions and shows good internal consistency for both the total score and the 2 subscales [108].

#### Assessment of Moderators

*Mobility* will be assessed using the corresponding subscale of the Patient Questionnaire for Medical Rehabilitation (Indicators of Rehabilitation Status-3) [109]. Respondents rate 4 items on a 5-point scale about how many difficulties they experienced during the past 4 weeks (eg, climbing a staircase over 3 floors). Items are rated on a 5-point scale from 1 (*impossible*) to 5 (*no difficulties*). The mobility subscale shows good internal consistency (Cronbach α=.85) [109].

*Attachment style* will be measured using the Adult Attachment Scale (AAS) [110,111]. The AAS comprises 16 items forming three subscales: (1) comfortability with closeness and intimacy, (2) the degree to which one can depend on others when they are needed, and (3) the degree to which one is worried about being rejected or unloved. The items are rated on a 5-point scale ranging from 1 (not at all characteristic of me) to 5 (very characteristic of me). The AAS shows satisfactory internal consistency (Cronbach α=.72-.79) [110].

*Childhood trauma* will be assessed using the 28-item Childhood Trauma Questionnaire [112,113]. Respondents are asked about their experiences of sexual, physical, and emotional maltreatment and physical and emotional neglect in childhood and adolescence. Items are rated on a 5-point Likert scale ranging from 1 (never true) to 5 (very often true). Higher values indicate a higher degree of childhood maltreatment. All scales (except physical neglect) show high internal consistency (Cronbach α ≥.89) [112].

### Sample Size

A power analysis was conducted using G*Power 3 [114]. We aim to detect small effect sizes [115] of 𝑓=0.10 (equivalent to Cohen *d*=0.20) regarding the time×group interaction for the 2 active conditions at an α error level of .05. A power analysis revealed that a sample size of 80 participants in each of the active study arms is required to detect a statistically significant difference with a power (1 – β) of 0.80, assuming correlations of *r*=0.60 between before and posttreatment measures, as previously found in another trial on ICBT for loneliness [57]. Sample size was further estimated based on a dropout rate of approximately 25%. We finally decided to randomize 100 participants to each of the active conditions. For the comparisons between the active conditions and the wait-list, 50 participants were considered sufficient for the wait-list control group condition as between-group effect sizes were assumed to be large based on the results of the aforementioned Swedish trials [54,57]. Consequently, we aim to randomize 250 participants with a randomization ratio of 2:2:1.

### Statistical Analyses

Analyses will be conducted using an intention-to-treat sample. In addition, we will conduct analyses in the per-protocol sample (ie, comprising participants who complete both baseline and postassessment measures and log into ≥4 modules of the program, defined as minimal therapeutic exposure). To assess if randomization was successful, we will compare baseline characteristics between the 3 conditions using chi-square tests (categorical) and *F* tests (continuous). Continuous outcomes will be analyzed using mixed-effects models. These analyses will model random slopes and intercepts for participants and test the fixed effects of the condition, as well as repeated assessments over time, using data from all participants. Differential intervention efficacy shows in significant interactions between the condition and time. An advantage of mixed-effects models is their ability to account for missing values through maximum likelihood estimation [116]. Significant overall effects will be followed up with contrasts comparing the 2 active conditions pooled together (ie, ICBT with and without guidance) against the control group, followed by a comparison of the 2 active conditions against each other. Significance levels will be set at P=.05. Effect sizes will be computed based on the work by Cohen [117], dividing the treatment effect by the pooled SD. In addition, we will calculate reliable changes according to the Reliable Change Index [118].

To test the mediation hypotheses, we will determine the extent of mediation of the change scores from baseline to week 5 (midassessment) and baseline to week 10 (postassessment) on the potential mechanism of change in week 10 and follow-up loneliness scores, respectively. Indirect effects will be tested by calculating bootstrapped CIs [119].

To test the moderation hypotheses, we will exploratorily include the scores of the various measures as the moderation variable to build 3-way interaction terms (condition×time×moderator) in separate mixed models. To facilitate the post hoc interpretation of interaction effects, continuous moderator variables will be grand mean centered [120]. Significant interaction effects will be followed up by applying the Johnson-Neyman technique [121].

## Results

Recruitment started in May 2021 and is expected to be completed in 2022, with the 12-month follow-ups to be completed in 2023. We intend to submit the first results for open access publication in 2023. In addition, the findings of this trial will be presented at national and international conferences. Only aggregated group data will be reported, and no individuals will be identifiable.

## Discussion

### General Discussion

Chronic loneliness is a prevalent clinical phenomenon in the population, with a variety of adverse effects on mental and physical health [8], leading to increased mortality [9]. Psychological interventions have generally been shown to be effective in reducing loneliness [48]. Such interventions at an individual level may play a crucial role in reducing the burden of loneliness and complementing social and societal-based interventions [122-124]. Although various treatments for alleviating loneliness have been developed and tested, more well-controlled clinical trials are needed to inform about efficacious interventions for loneliness.

This study aims to test the efficacy of an internet-based self-help intervention (*SOLUS-D*) in reducing loneliness. We expect participants in both intervention groups to show greater decreases in loneliness compared with a wait-list control group at the posttreatment time point. Furthermore, reductions in loneliness will be expected to be more pronounced in participants receiving guidance than in the group without guidance. By comparing the 2 intervention groups with and without guidance, we will be able to inform about the incremental effect of guidance beyond that of the ICBT intervention. We will also look at acceptability aspects such as intervention satisfaction, adherence, and potential negative effects to further refine the intervention. In addition, we will investigate potential moderating variables on an exploratory level to gain insights into how and for whom exactly the intervention works. Furthermore, knowledge on mechanisms of change in treating chronic loneliness will be expanded. This will provide further insight into the maintaining factors of chronic loneliness that interventions should focus on and, at the same time, test the proposed model of chronic loneliness [19]. Finally, the results of this study will inform which individuals can profit (most) from an internet-based ICBT intervention for loneliness.

### Limitations

The limitations of our study must be considered. First, the sample in our study is self-selective and excludes individuals with no access to the internet or a device to use the intervention. Second, people with heightened degrees of symptom severity of mental disorders, such as at least moderately severe depressive symptoms, will be excluded from this study. Consequently, results will not be generalizable to all people experiencing loneliness. Third, within our study design, we will not be able to compare the long-term effects of the intervention with the wait-list, as we did not want the wait-list control group to wait too long to have access to the intervention. Fourth, we did not want to restrict access to care as usual in this study. This reduces the internal validity of this study to some extent while simultaneously increasing its external validity.

### Conclusions

In this study protocol, we describe the design of an RCT evaluating an internet-based self-help intervention with and without guidance based on CBT principles for loneliness compared with a wait-list control group in adults. To the best of our knowledge, this is the first RCT to evaluate an internet-based self-help intervention based on CBT principles for loneliness in German-speaking countries. The results of this study will enrich previous findings on the efficacy of internet-based self-help interventions for loneliness and inform on what treatments work for whom in alleviating loneliness. On the basis of our findings, policy makers could be informed about the efficacy of interventions with low-threshold access for alleviating loneliness.

## Abbreviations

AAS: Adult Attachment Scale
CBT: cognitive behavioral therapy
DSM: Diagnostic and Statistical Manual of Mental Disorders
ICBT: internet-based cognitive behavioral therapy
ICD: International Classification of Diseases
IIPT: internet-based interpersonal therapy
INEP: Inventory for the Assessment of Negative Effects of Psychotherapy
KGAI: Kernis-Goldman Authenticity Inventory
Mini-DIPS: Mini Diagnostic Interview for Mental Disorders
PHQ-9: Patient Health Questionnaire-9
RCT: randomized controlled trial
UCLA: University of California, Los Angeles
WAI-I: Working Alliance Inventory for Internet interventions
